# HDL-Associated Proteins in Subjects with Polycystic Ovary Syndrome: A Proteomic Study

**DOI:** 10.3390/cells12060855

**Published:** 2023-03-09

**Authors:** Alexandra E. Butler, Abu Saleh Md Moin, Željko Reiner, Thozhukat Sathyapalan, Tannaz Jamialahmadi, Amirhossein Sahebkar, Stephen L. Atkin

**Affiliations:** 1 Research Department, Royal College of Surgeons in Ireland Bahrain, Adliya 15503, Bahrain; 2 Department of Internal Medicine, University Hospital Center Zagreb, Kišpatićeva 12, 10000 Zagreb, Croatia; 3 Academic Endocrinology, Diabetes and Metabolism, Hull York Medical School, Hull HU6 7RX, UK; 4 Applied Biomedical Research Center, Mashhad University of Medical Sciences, Mashhad 91778, Iran; 5 Biotechnology Research Center, Pharmaceutical Technology Institute, Mashhad University of Medical Sciences, Mashhad 91778, Iran; 6 School of Medicine, The University of Western Australia, Perth, WA 6009, Australia; 7 Department of Biotechnology, School of Pharmacy, Mashhad University of Medical Sciences, Mashhad 91778, Iran

**Keywords:** HDL, lipoprotein, obesity, PCOS, proteomics, slow off-rate modified aptamer

## Abstract

Introduction. Serum lipoproteins, with the exception of high-density lipoprotein cholesterol (HDL-C), are increased in polycystic ovary syndrome (PCOS) and their levels may reflect the associated obesity and insulin resistance, but the nature of this association is not fully explained. Therefore, proteomic analysis of key proteins in lipoprotein metabolism was performed. Methods. In this cohort study, plasma was collected from 234 women (137 with PCOS and 97 controls without PCOS). Somalogic proteomic analysis was undertaken for the following 19 proteins involved in lipoprotein, and particularly HDL, metabolism: alpha-1-antichymotrypsin; alpha-1-antitrypsin; apolipoproteins A-1, B, D, E, E2, E3, E4, L1, and M; clusterin; complement C3; hemopexin; heparin cofactor II; kininogen-1; serum amyloid A-1; amyloid beta A-4; and paraoxonase-1. Results. The levels of apolipoprotein E were higher in PCOS (*p* = 0.012). However, the other isoforms of ApoE, ApoE2, E3, and E4, did not differ when compared with controls. ApoM was lower in PCOS (*p* = 0.000002). Complement C3 was higher in PCOS (*p* = 0.037), as was heparin cofactor II (HCFII) (*p* = 0.0004). The levels of the other proteins associated with lipoprotein metabolism did not differ between PCOS and controls. Conclusions. These data contribute to the concern of the deleterious dyslipidemia found in PCOS, with the novel combination reported here of higher levels of ApoE, C3 and HCFII together with lower ApoM. The dysregulation of these proteins could circumvent the protective effect of HDL-C and contribute to a more atherogenic profile that may increase cardiovascular risk.

## 1. Introduction

Polycystic ovary syndrome (PCOS) is the most common endocrine disorder in premenopausal women, with its prevalence reported as 10–15% [1]. It is associated with an increased prevalence of type 2 diabetes, hypertension, insulin resistance, metabolic syndrome, and potentially cardiovascular disease (CVD) [2]. This may be mediated by inflammation, although the underlying pathophysiological mechanism remains unclear [3]. PCOS is also associated with nonalcoholic fatty liver disease (NAFLD) [4] and nonalcoholic steatohepatitis [5] and these diseases are associated with dyslipidemia and CVD. Dyslipidemia, the most important risk factor for CVD, is common in young adult women with PCOS [6,7]. In particular, the role of high-density lipoprotein cholesterol (HDL-C) particles is critical because decreased cholesterol efflux capacity has been shown in young women with PCOS, which increases their risk of atherosclerosis and CVD [8]. Both retrospective and prospective cohort studies report increased atherosclerotic coronary heart disease (ASCHD) in women with PCOS [9]. 

**Figure 1:**
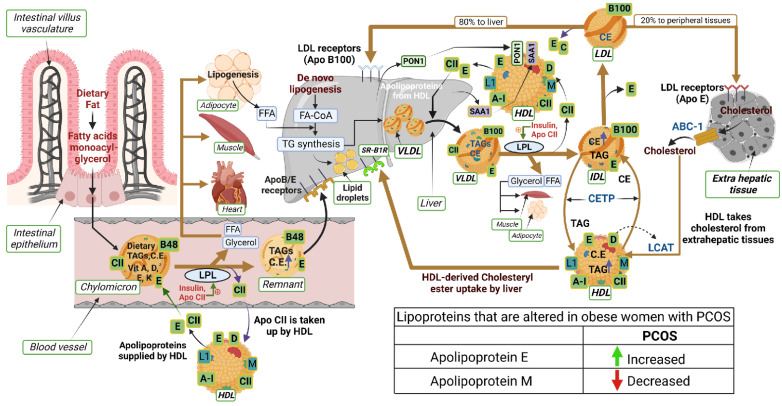
Schematic illustrating the interactions of proteins and lipids in the pathways of lipoprotein metabolism. The lipoproteins that are changed in obese subjects with PCOS are indicated in the table (bottom right in the illustration). An upward green arrow indicates an increase and a downward red arrow indicates a decrease in lipoproteins in PCOS.

Apolipoproteins are the main components determining the metabolic fate of serum lipoprotein particles, as well as other proteins involved in lipoprotein metabolism (Figure 1). Dietary fats (exogenous triglycerides) are carried by nascent chylomicrons synthesized by the intestine. Chylomicrons are assembled in the intestinal mucosal cells and carry dietary triacylglycerol (TAGs), cholesterol, fat soluble vitamins (vitamin A, D, E, and K), and cholesteryl esters (CEs). TAGs account for close to 90% of the lipids in a chylomicron. Chylomicrons are hydrolyzed by an extracellular enzyme, lipoprotein lipase (LPL), which is anchored by heparan sulphate to the capillary wall of most tissues. LPL is activated by insulin and apolipoprotein CII (CII) on circulating chylomicrons and hydrolyzes the TAGs to yield free fatty acids (FFAs) and glycerol. In muscle, fatty acids are oxidized for energy; in adipose tissue, they are re-esterized as TAGs for storage. Upon being hydrolyzed by LPL, a chylomicron is converted into a chylomicron remnant (which contains lipoproteins, Apo B-48, and Apo E). Chylomicron remnant (Apo B-48, Apo E) enters the liver through the low-density lipoprotein (LDL) receptor (Apo B-100, E). Apo CII from chylomicrons is taken back by HDL. De-novo synthesis of fatty acids (endogenous triglycerides) in the liver is carried out by nascent very-low-density lipoprotein (VLDL) (containing lipoproteins Apo B-100, Apo CII, and Apo E). Lipoprotein lipase hydrolyzes TAG to FFAs and the remnant of VLDL is called intermediate density lipoprotein IDL (containing Apo B-100, Apo E), which has lost triglyceride, but is rich in cholesterol. IDL or VLDL remnant (B-100, E) has two fates: largely, it loses Apo-E and is converted to LDL (B-100) and, minorly, it is taken up by liver via the LDL receptor. LDL (B-100) has two fates: 80% of LDL enters the liver through the LDL receptor (ApoB-100, E), while 20% enters extrahepatic tissues through the LDL receptor (ApoE). HDL acts as a reservoir for different apoproteins and exchanges them with other lipoproteins. It provides Apo CII and E to nascent chylomicrons and VLDL to form chylomicron (B-48, CII, E) and VLDL (B-100, CII, E). Besides being a reservoir for apoproteins, it serves the function of reverse cholesterol transport. HDL takes cholesterol from extrahepatic tissues through the ATP binding cassette (ABC-1) transporter. Lectin-cholesterol acyltransferase (LCAT) in HDL (stimulated by A-1 and Apo D) converts cholesterol to cholesteryl ester. Cholesteryl ester transport protein (CETP) mediates the exchange of cholesteryl ester for triglyceride from HDL with other lipoproteins. Reuptake of HDL-derived C occurs in the liver through scavenger receptor class B type 1 (SR-B1). The other apoproteins of HDL involve Apo L1 and Apo M. Two other HDL-associated proteins, paraoxonase-1 (PON1) and serum amyloid A1 (SAA1), are synthesized in the liver and later associated with the HDL moiety.

**Figure 2:**
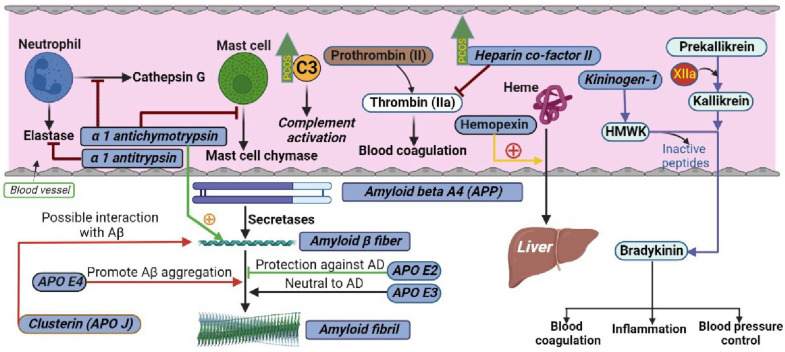
Schematic illustrating the interactions of proteins measured in the current study in their respective biological pathways. The levels of C3 and heparin cofactor II were higher in PCOS, as indicated by green upward arrows.

Alzheimer’s disease (AD)-related protein amyloid beta (Aβ) has also been reported to interact with normal human plasma HDL [10]. Amyloid beta A4, also known as amyloid precursor protein (APP), functions as a cell surface receptor and transmembrane precursor protein, which is cleaved by secretases to form amyloid beta (Aβ) fiber. Human APOE lipoprotein isoforms, APOE2, APOE3, and APOE4, are involved in the pathobiology of AD. While APOE2 has a protective effect against amyloid fibril formation, APOE3 has no effect in amyloid fibril generation. APOE4, which promotes Aβ aggregation, constitutes the most significant genetic risk factor for AD. APOJ (also known as clusterin) has also been found to positively interact with Aβ (Figure 2). The regulatory proteins of Aβ, α-1 antichymotrypsin (ACT), a plasma serine protease inhibitor, targets neutrophil cathepsin G as well as mast cell chymase. ACT is an integral component of the amyloid deposits in Alzheimer’s disease (AD) and had been shown to catalyze amyloid beta (Aβ) polymerization. Alpha 1 antitrypsin (A1AT) is a serum proteinase inhibitor, especially for neutrophil elastase. While women with PCOS have shown worse cognitive performance compared with non-PCOS women across domains of memory, executive function, and brain structure [11], the association between HDL- and AD-related proteins in PCOS has not been explored before.

HDL acts as an anticoagulant and its antithrombotic property involves modulation of platelet reactivity and endothelial function [12]. Kininogen-1 (encoded by the KNG1 gene in humans) is a α-2-thiol proteinase inhibitor and a constituent of the blood coagulation system as well as the kinin-kallikrein system. KNG1 gene undergoes alternative splicing to generate high-molecular-weight kininogen (HMWK) and low-molecular-weight kininogen. HMWK in turn is cleaved by the enzyme kallikrein (synthesized by pre-kallikrein with the help of coagulation factor XIIa) to produce bradykinin (Figure 2). Bradykinin is a potent endothelium-derived vasodilator and is involved in many biological processes including blood coagulation, inflammation, and blood pressure control [13]. As the risk of developing hypertension in young women with PCOS is higher than in controls, the role of proteins in the kinin-kallikrein system has not been elucidated in PCOS.

An inverse association has been reported between high-density lipoprotein cholesterol (HDL-C) and the risk of atherosclerotic coronary artery disease (ASCAD) in multiple clinical and epidemiological studies [14,15], indicative of the protective effect of HDL-C. Accordingly, low levels of HDL-C underlie the most frequent form of familial dyslipidemia in younger patients with myocardial infarction [16]. In prospective studies, low levels of HDL-C and high levels of C-reactive protein were independent factors elevating the risk of a second event in patients with known ASCAD [17,18]. Furthermore, in a large interventional trial, elevating the level of HDL-C was shown to decrease the incidence of ASCAD [19]. The protective effects of HDL-C on ASCAD are considered to be a consequence of HDL-C’s role in reverse cholesterol transport, removing cholesterol from the periphery to the liver for processing and excretion in the bile [20]. However, further evidence suggests that HDL-C additionally exerts a protective influence on endothelial function [21].

As PCOS is associated with CVD, determining the levels of serum lipoproteins in PCOS subjects is important. Therefore, the aim of this study was to utilize state-of-the-art proteomics to determine circulating levels of proteins specifically involved in lipoprotein metabolism, particularly of HDL, in subjects with PCOS.

## 2. Materials and Methods

Plasma levels of proteins involved in lipoprotein, and particularly HDL, metabolism were measured in women with (n = 137) and without PCOS (n = 97) from a PCOS biobank (ISRCTN70196169: 2012–2017, approved by the Newcastle and North Tyneside Ethics Committee [22]); all subjects provided written informed consent.

The women were all Caucasian [22], with PCOS diagnosed according to the Rotterdam consensus, based on two out of three of the criteria; namely, clinical and biochemical evidence of hyperandrogenism (Ferriman–Gallwey score > 8), free androgen index > 4 (total testosterone > 1.5 nmol/L), and oligomenorrhea or amenorrhoea and polycystic ovaries diagnosed by transvaginal ultrasound. Confounding diagnoses such as nonclassical 21-hydroxylase deficiency were appropriately screened in detail previously [22]. The demographic data for the PCOS and control cohorts are shown in Table 1 [22]. Controls had regular menses, normal physical examination, and polycystic ovaries excluded by ultrasound, and were not on any medications.

Blood was withdrawn fasting and prepared by centrifugation at 3500× *g* for 15 min, aliquoted, and stored at −80 °C. Analysis for sex hormone binding globulin (SHBG), insulin (DPC Immulite 200 analyser, Euro/DPC, Llanberis, UK), and plasma glucose (Synchron LX20 analyser, Beckman-Coulter, High Wycombe, UK) was undertaken. Free androgen index (FAI) was derived from total testosterone divided by SHBG ×100. Insulin resistance (IR) was calculated using the homeostasis model assessment (HOMA-IR). Serum testosterone was quantified using isotope-dilution liquid chromatography tandem mass spectrometry (LC-MS/MS) [23].

Plasma lipid-related proteins were measured by the slow off-rate modified aptamer (SOMA)-scan platform [24]. Calibration was based on standards as previously described [25].

SOMAscan technology offers significant advantages in sample size, cost, time, multiplexing capability, dynamic range, and flexibility of readout over many alternate protein biomarker platforms. The protein quantification was performed using a slow off-rate modified aptamer (SOMAmer)-based protein array, as previously described [26,27]. Briefly, EDTA plasma samples were diluted and the following assay steps were performed: (1) Binding—analytes and primer bead (PB)/SOMAmers (fully synthetic fluorophore-labeled SOMAmer coupled to a biotin moiety through a photocleavable linker) were equilibrated. (2) Catch I—all analyte/SOMAmers complexes were immobilized on a streptavidin-substituted support. Washing steps removed proteins not stably bound to PB/SOMAmers and the bound protein was biotinylated. (3) Cleave—long-wave ultraviolet light was applied to release analyte/SOMAmer complexes into the solution. (4) Catch II—analyte/SOMAmer complexes were selectively immobilized on streptavidin support via the introduced analyte-borne biotinylation. Further washing was continued to select against unspecific analyte/SOMAmer complexes. (5) Elution–denaturation caused disruption of analyte/SOMAmer complexes. Released SOMAmers serve as surrogates for the quantification of analyte concentrations. (6) Quantification–hybridization to custom arrays of SOMAmer-complementary oligonucleotides.

Normalization of raw intensities, hybridization, median signal, and calibration signal were performed based on the standard samples included on each plate, as previously described [24,25].

Version 3.1 of the SOMAscan assay, targeting those proteins specifically involved in lipoprotein and particularly HDL metabolism in the SOMAscan panel, was used. These 19 proteins were alpha-1-antichymotrypsin; alpha-1-antitrypsin; apolipoproteins A-1, B, D, E, E2, E3, E4, L1, and M; clusterin; complement C3; hemopexin; heparin cofactor II; kininogen-1; serum amyloid A-1; amyloid beta A-4; and paraoxonase-1 (PON1).

## 3. Statistics

Power was based on C3 protein changes reported to be different in PCOS [28] (nQuery version 9, Statsols, Boston, MA, USA). For an alpha of 0.05, with a common standard deviation (SD) of 0.37 based on 80% power, a total of 23 subjects per arm were required. Visual inspection of the data was undertaken followed by Student’s *t*-tests for normally distributed data and Mann–Whitney tests for non-normally distributed data, as determined by the Kolmogorov–Smirnov test. All analyses were performed using Graphpad Prism version 9.4.1 (San Diego, CA, USA).

## 4. Results

Baseline data for the 146 PCOS subjects and 97 controls are shown in Table 1. The two cohorts were age-matched, but subjects with PCOS had a greater BMI, had increased insulin resistance, hyperandrogenemia, and increased C-reactive protein (CRP, an inflammatory marker). Regarding the circulating lipoprotein profile, triglycerides (TGs) were higher (*p* = 0.001) and high-density lipoprotein cholesterol (HDL-C) was lower (*p* < 0.0001) in PCOS, while total cholesterol and low-density lipoprotein cholesterol (LDL-C) were comparable (*p* = 0.22 and 0.16, respectively). The TG/HDL-C ratio was higher in PCOS versus controls (*p* = 0.001).

**Figure 3:**
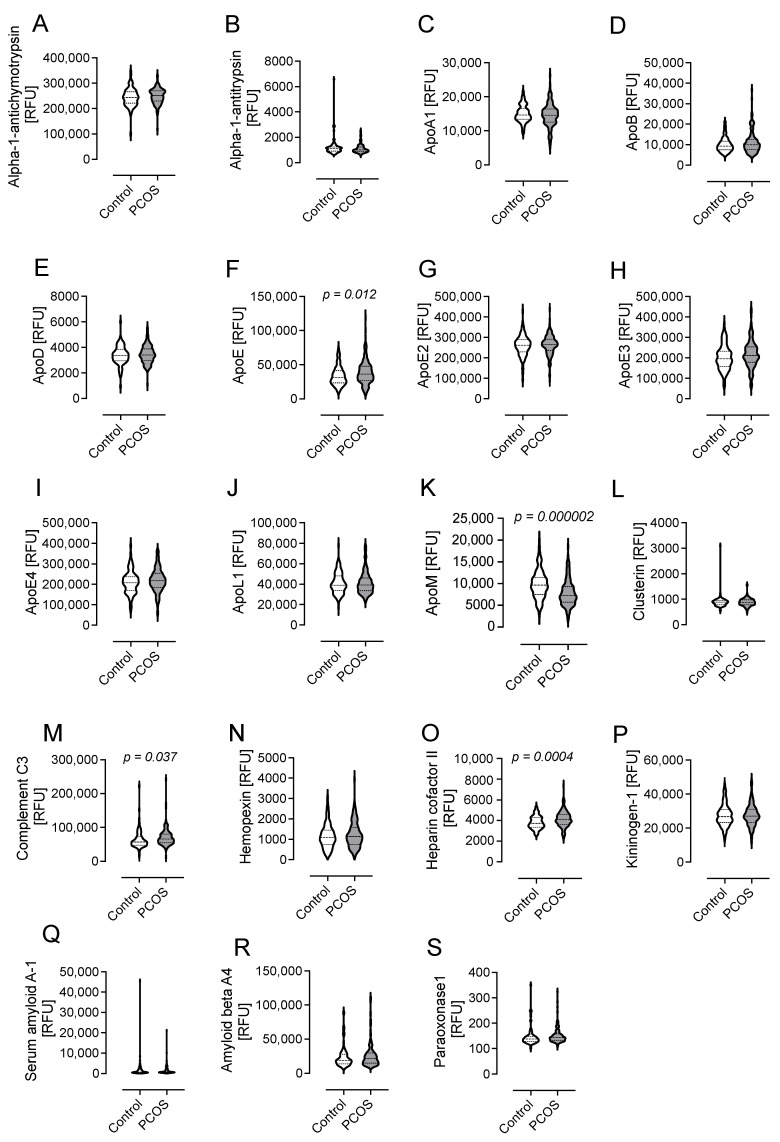
Levels of proteins involved in lipid metabolism in subjects with polycystic ovary syndrome (PCOS) fulfilling all three diagnostic criteria versus controls. Data presented as violin plots with mean ± 1 standard deviation of relative fluorescent units (RFUs). These 19 proteins were alpha-1-antichymotrypsin (**A**); alpha-1-antitrypsin (**B**); apolipoproteins A-1 (**C**), B (**D**), D (**E**), E (**F**), E2 (**G**), E3 (**H**), E4 (**I**), L1 (**J**), and M (**K**); clusterin (**L**); complement C3 (**M**); hemopexin (**N**); heparin cofactor II (**O**); kininogen-1 (**P**); serum amyloid A-1 (**Q**); amyloid beta A-4 (**R**); and paraoxonase-1 (**S**).

The results of the Somascan analysis of lipid-metabolism-related proteins are shown as violin plots in Figure 3 and numerically in Supplementary Table S1 for PCOS subjects and control subjects.

### 4.1. Levels of Proteins Involved in Lipid Metabolism in PCOS

The levels of apolipoprotein E (ApoE) were higher in PCOS (39,054 ± 17,973 vs. 33,577 ± 13,945 RFU, *p* = 0.012, PCOS vs. control). However, the isoforms of ApoE-ApoE2, E3, and E4- were not different in PCOS compared with women without PCOS (26,1934 ± 50,357 vs. 259,099 ± 51,595 RFU of ApoE2, *p* = 0.67, PCOS vs. control; 217,377 ± 67,477 vs. 201,576 ± 61,141 RFU of ApoE3, *p* = 0.06, PCOS vs. control; 219,789 ± 58305 vs. 210,604 ± 55,689 RFU of ApoE4, *p* = 0.22, PCOS vs. control).

ApoM was lower in PCOS (7878 ± 3039 vs. 9868 ± 3277 RFU, *p* = 0.000002, PCOS vs. control). 

Complement C3 (C3) was higher in PCOS (71,028 ± 25,536 vs. 63,896 ± 26,822 RFU of C3, *p* = 0.037, PCOS vs. control), as was heparin cofactor II (HCFII) (4156 ± 773 vs. 3821 ± 618 RFU of HCFII, *p* = 0.0004, PCOS vs. control) (Figure 3 and Supplementary Table S1).

The levels of other proteins associated with lipid metabolism, namely, alpha-1-antichymotrypsin; alpha-1-antitrypsin; apolipoproteins A-1, B, D, E2, E3, E4, and L1; clusterin; hemopexin; kininogen-1; serum amyloid A-1; amyloid beta A-4; and paraoxonase-1, were comparable between PCOS subjects and controls (Figure 3 and Supplementary Table S1).

### 4.2. Correlation Analyses

For the four proteins that differed between PCOS subjects and control women (ApoE, ApoM, C3, and HCFII), correlations with age; BMI; insulin resistance (HOMA-IR); testosterone; and circulating levels of TG, cholesterol, HDL-C, LDL-C, and CRP were performed. 

**Figure 4:**
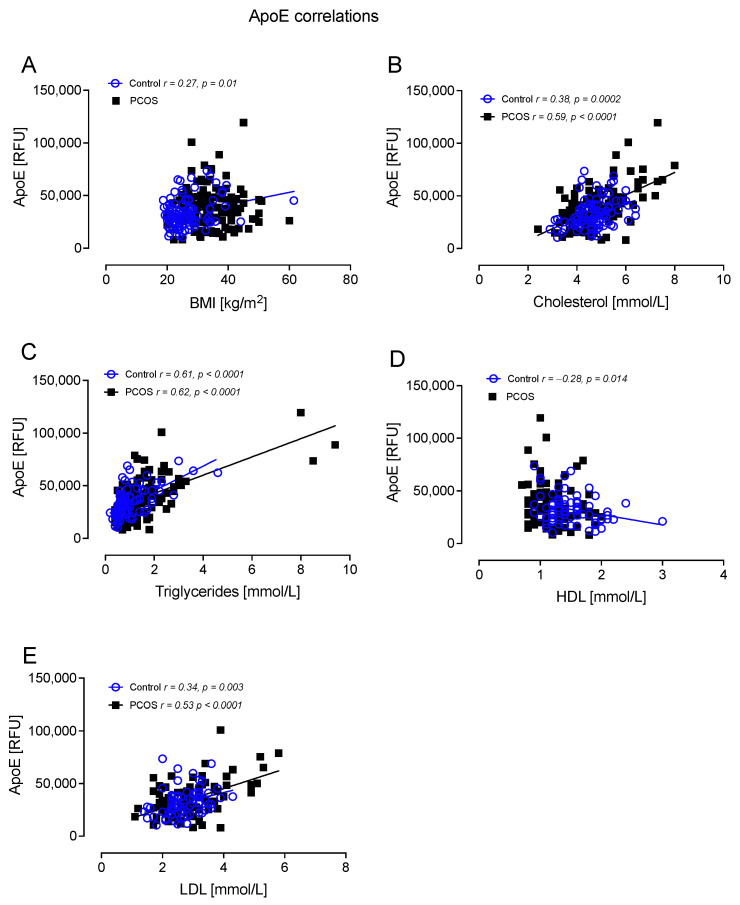
ApoE correlated significantly with BMI in controls (**A**), cholesterol in controls and PCOS (**B**), triglycerides in controls and PCOS (**C**), HDL in controls (**D**), and LDL in controls and PCOS (**E**).

ApoE correlated positively with BMI in controls (r = 0.27, *p* = 0.01); correlated positively with total cholesterol in controls (r = 0.38, *p* = 0.0002) and PCOS (r = 0.59, *p* < 0.0001); correlated positively with TG in controls (r = 0.61, *p* < 0.0001) and PCOS (r = 0.62, *p* < 0.0001); correlated negatively with HDL-C in controls (r = −0.28, *p* = 0.014); and correlated positively with LDL-C in controls (r = 0.34, *p* = 0.003) and PCOS (r = 0.53 *p* < 0.0001) (Figure 4).

**Figure 5:**
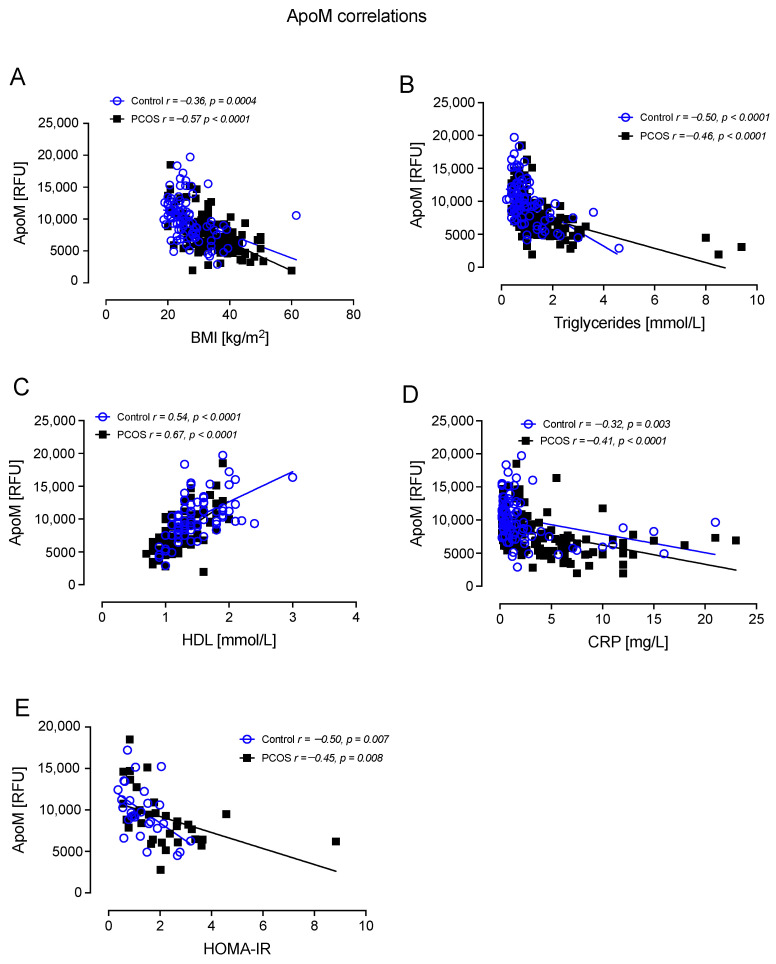
ApoM correlated significantly with BMI in controls and PCOS (**A**), triglycerides in controls and PCOS (**B**), HDL in controls and PCOS (**C**), CRP in controls and PCOS (**D**), and HOMA-IR in controls and PCOS (**E**).

ApoM correlated negatively with BMI in controls (r = −0.36, *p* = 0.0004) and PCOS (r = −0.57, *p* < 0.0001); correlated negatively with TG in controls (r = −0.50, *p* < 0.0001) and PCOS (r = −0.46, *p* < 0.0001); correlated positively with HDL-C in controls (r = 0.54, *p* < 0.0001) and PCOS (r = 0.67, *p* < 0.0001); correlated negatively with CRP in controls (r =−0.32, *p* = 0.003) and PCOS (r = −0.41, *p* < 0.0001); and correlated negatively with HOMA-IR in controls (r = −0.50, *p* = 0.007) and PCOS (r = −0.45, *p* = 0.008) (Figure 5).

**Figure 6:**
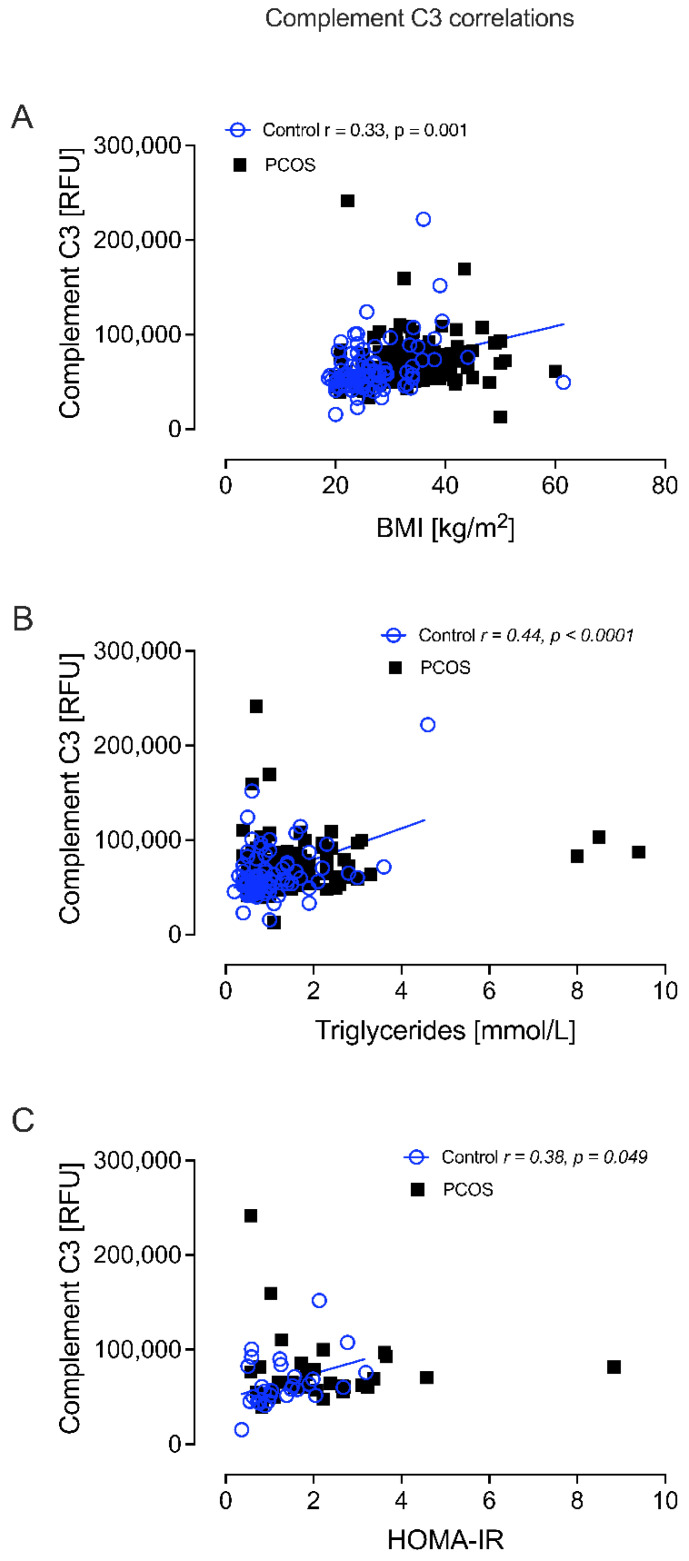
Complement C3 correlated significantly with BMI in controls (**A**), triglycerides in controls (**B**), and HOMA-IR in controls (**C**).

Complement C3 correlated positively with BMI in controls (r = 0.33, *p* = 0.001); correlated positively with TG in controls (r = 0.44, *p* < 0.0001); and correlated positively with HOMA-IR in controls (r = 0.38, *p* = 0.049) (Figure 6).

**Figure 7:**
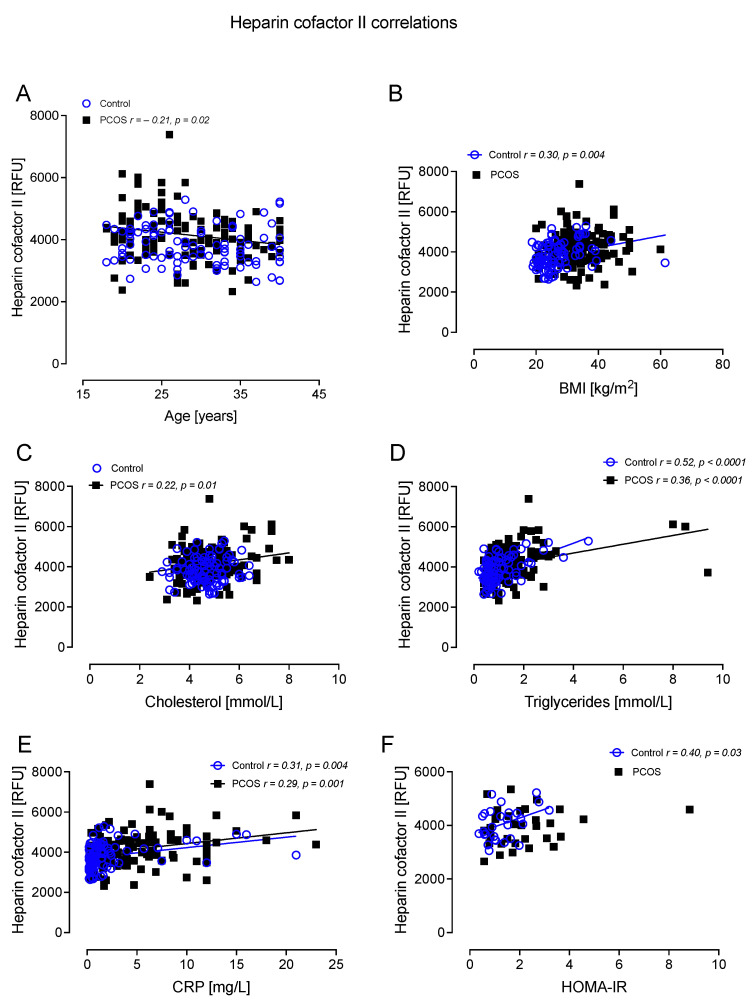
Heparin cofactor II (HCFII) correlated significantly with age in PCOS (**A**), BMI in controls (**B**), cholesterol in PCOS (**C**), triglycerides in controls and PCOS (**D**), CRP in controls and PCOS (**E**), and HOMA-IR in controls (**F**).

HCFII correlated negatively with age in PCOS (r =−0.21, *p* = 0.02); correlated positively with BMI in controls (r = 0.30, *p* = 0.004); correlated positively with cholesterol in PCOS (r = 0.22, *p* = 0.01); correlated positively with TG in controls (r = 0.52, *p* < 0.0001) and PCOS (r = 0.36, *p* < 0.0001); correlated positively with CRP in controls (r = 0.31, *p* = 0.004) and PCOS (r = 0.29, *p* = 0.001); and correlated positively with HOMA-IR in controls (r = 0.40, *p* = 0.03) (Figure 7).

## 5. Discussion

The results presented here show that the levels of ApoE (but not the isoforms ApoE2, ApoE3, and Apo E4), complement C3, and heparin cofactor II were higher, while the levels of ApoM were lower in women with PCOS versus control women.

Several studies have been performed on women with PCOS analyzing their serum lipoproteins. One most recently published retrospective study on 700 Chinese women indicated that most of these subjects had low HDL-C; that subjects with clinical hyperandrogenism also had lower ApoA levels; and that the levels of TG, LDL-C, and ApoB were increased in women with PCOS with insulin resistance. This study also showed that TG and ApoB levels showed a trend towards an increase with BMI and that ApoAI, TG/HDL-C, and ApoB/ApoA ratios were linked to certain features of PCOS, specifically insulin resistance and obesity [29]. However, these authors did not compare women with PCOS to healthy women, neither did they perform a proteomic study. In one of the earliest studies on PCOS and dyslipidemia performed on south-west Chinese women with PCOS, now published a decade ago, HDL-C was decreased and TG was increased in women with PCOS versus women without PCOS. PCOS subjects who exhibited dyslipidemia had not only higher TG/HDL-C ratios, but also lower HDL-C and ApoAI levels versus controls or PCOS subjects without dyslipidemia. They also had higher BMI and fasting and 2 h insulin and glucose concentrations, as well as increased homeostatic model assessment IR (HOMA-IR); atherogenic indexes; TG, LDL-C, and ApoB concentrations; and ApoB/ApoA-I ratios versus controls [30].

The association between Apo E, and particularly ApoE2 (one of the three isoforms of the *APOE* gene), and atherogenesis is still controversial, although evidence suggests that apolipoprotein gene rs7412 (E2) and rs429358 (E4) single nucleotide polymorphisms (SNPs) may be associated with CVD risk. The results of a cohort study of south-west Chinese women showed no associations of any ApoE genotype with PCOS, and accord with the results of the present study [31]. In a study on subjects with PCOS in Western Anatolia, Turkey, the ApoE3 allele was reported at a higher frequency in PCOS subjects versus controls, although no significant difference was found in lipid or other CVD risk factors with regard to allele and genotype data [32]. Another study reported similar findings in PCOS with no significant difference in E3, E4, and E2 alleles of ApoE genes [33]. Of note, higher apoE in whole plasma and a higher apoE content in HDL particles were associated with lower dementia risk [34] and, conversely, reduced plasma ApoE plus the *APOE* ε4 allele were associated with elevated risk for Alzheimer’s disease [35]. Polymorphism of the ApoE gene is a major risk for Alzheimer’s disease, with the strongest risk being for the ε4 allele associated with lower levels of ApoE, while the ε2 allele is protective with higher levels of ApoE [36]. The various ApoE isoforms differ in their ability to bind lipids and amyloid-β, a critical protein in Alzheimer’s disease [36]. This finding of increased levels of ApoE may be pertinent in protecting against the potential increased risk of Alzheimer’s disease in PCOS, where it has been shown that these subjects may share some common risk factors [37].

Here, no association of PCOS and ApoB was found, though ApoB showed a trend to increase; this result differs from a study of young girls with PCOS, where elevated plasma apoB48-lipoprotein remnants were found to be highly associated with cardiometabolic risk and had ~2-fold elevated prevalence compared with girls without PCOS. Therefore, this may predispose them to prematurely developing atherosclerosis and CVD [38].

A recent study including proteomics of potential insulin resistance biomarkers in PCOS women showed that only ApoC3 was flagged as potentially being a diagnostic marker for PCOS-insulin-resistant subjects [39]; however, in this study, ApoC3 was not available in the Somascan panel. A single nucleotide polymorphism with a reduction in ApoM transcriptional activity and a decrease in serum ApoM levels has been reported as a potential biomarker for coronary artery disease [40]. Decreased ApoM has not been reported in PCOS previously and, in this context, may contribute to the increased atherogenic dyslipidemia seen in PCOS.

ApoM is an apolipoprotein primarily located in HDL particles and required in their formation and HDL-mediated reverse cholesterol transport. ApoM is linked to the anti-atherosclerotic, anti-inflammatory, and anti-oxidant effects of HDL particles, and is associated with several diseases, including ASCVD [41]. It is interesting that plasma ApoM levels are low in subjects with type 2 diabetes mellitus (T2DM), a phenomenon that, according to some authors, is likely caused by diabetes and is not a consequence of the dyslipidemia that often accompanies T2DM [42]. However, others consider that no causal association exists between plasma ApoM and an elevated risk of T2DM [43]. ApoM, the major plasma carrier of the bioactive lipid mediator sphingosine-1-phosphate (S1P), seems to underlie several HDL-associated protective functions in the endothelium, including the regulation of adhesion molecule quantity, leukocyte–endothelial adhesion, and the endothelial barrier limiting endothelial inflammation by delivering S1 to the S1P receptor 1 [44]. Lower levels of ApoM, as found in this study, probably lead to lower ApoM/S1 levels. It has been suggested that the ApoM/S1 complex has a protective role against the development of insulin resistance, a common feature of PCOS [45]; therefore, lower ApoM levels may contribute to the insulin resistance commonly seen in PCOS and that is an independent cardiovascular risk marker [46]. Others have reported that single nucleotide polymorphisms with a reduction in ApoM transcriptional activity and a decrease in serum ApoM levels may be biomarkers for coronary artery disease [40]. Cardiac insulin resistance generates damage by at least three different mechanisms, including signal transduction alteration, impaired regulation of substrate metabolism, and altered delivery of substrates to the myocardium [40].

Higher levels of complement C3 were found here in PCOS and have been reported previously [47], which may reflect an association with the higher risk of obesity and atherosclerosis. In a PCOS population, complement C3 was associated with coronary artery calcification [48]. HDL subspecies that contain C3 are associated with higher CHD risk versus HDL without complement C3 [49]. One possible explanation might be that complement C3 is associated with ApoC2 and indirectly with ApoE [50]. The complement C3 fragment, C3a-desArg, acts as a hormone with insulin-like effects and aids triglyceride metabolism, but also promotes the production of inflammatory initiators such as the anaphylatoxin C3a, potentiating atherogenesis [51]. It has been shown that complement C3 is linked to an adverse lipoprotein profile, featuring increased triglyceride-enriched lipoproteins though fewer large HDL particles [52]. In addition to elevated triglycerides, lower levels of large HDL particles seem to be linked with increased CHD risk [53,54]. Increased levels of complement C3 have a greater association with insulin resistance than markers of inflammation such as C-reactive protein [55], and a recent study has shown a strong association of serum complement C3 with serum insulin and insulin resistance in subjects with PCOS, suggesting that this inflammatory marker might predict future diabetes and CVD complication risk in subjects with PCOS [56]; therefore, it is not surprising that, in this study, complement C3 was also elevated in PCOS versus controls. This also suggests that increased insulin resistance is mediated by complement C3 rather than complement C3, being an epiphenomenon of inflammation. In accord with the increased insulin resistance associated with reduced ApoM noted above, the increased complement C3 may augment the insulin resistance seen and its associated cardiovascular risk.

Studies suggest that high plasma HCII levels are protective against in-stent restenosis and atherosclerosis [57], with HCII deficiency promoting atherogenesis in mice [58], indicative of the important role that HCII may play in vascular homeostasis. Higher heparin cofactor II (HCFII) levels were found in PCOS; however, there is scant literature on the role of HCFII in the metabolism of serum lipoproteins and development of atherogenesis. In a single study, heparin cofactor II activity and HDL-C were negatively correlated with maximum atherosclerotic plaque thickness; plasma heparin cofactor II activity and HDL-C concentration independently contributed to plaque thickness, with the antiatherogenic effects of heparin cofactor II activity being greater than the effect of HDL-C [59]. It has been reported that the HCII level was negatively correlated with a higher vulnerability of carotid plaques and that plasma HCII may be a potential biomarker for the evaluation of the vulnerability of carotid plaques [60]. Thus, in this study, the high levels of HCII may mitigate and protect against the potential detrimental effects of the raised complement C3 and the low ApoM.

In this study, we did not find a decrease in PON1 activity (an antioxidant that serves to prevent lipoprotein oxidation and to hydrolyze atherogenic products generated from oxidative lipid modification). Such a decrease was, however, found in a meta-analysis of PCOS women [61]. Limited studies have suggested that antioxidant therapy in PCOS, such as with alpha lipoic acid, improves oxidative stress and insulin resistance, promotes follicular maturation, and improves glucose and lipid metabolism and vascular endothelial function, though robust and sufficiently powered studies are necessary to confirm these findings [62].

Serum complement C3 was reported to have a stronger link with insulin resistance than with high-sensitivity C-reactive protein (hsCRP) in PCOS women [55]. Therefore, it is not surprising that, in this study, complement C3 was also elevated in PCOS versus controls. A recent study has shown a strong association of serum complement C3 with serum insulin and insulin resistance in subjects with PCOS, suggesting that this inflammatory marker might predict future diabetes and CVD complication risk in subjects with PCOS [56].

The level of heparin cofactor-II in subjects with PCOS was shown to be increased [63]; however, in a multivariate analysis where BMI, inflammation, and insulin resistance were accounted for, no correlation of PCOS with either heparin cofactor II or any coagulation proteins was seen, suggesting that hypercoagulability is not an intrinsic facet of PCOS. Thus, PCOS should not be considered as a risk factor on the Veno Thrombo embolism risk assessment unless associated with other risk factors such as obesity, hormonal stimulation, or smoking.

The limitations of this study include that all participants were Caucasian and, therefore, the findings may differ, to a greater or lesser extent, in other ethnic groups. Moreover, protein measurements are reported as relative fluorescent units (RFUs) by the SOMAscan software version 4.1 and cannot be converted to protein concentrations.

In conclusion, these data contribute to the concern of the deleterious dyslipidemia found in women with PCOS, with the novel combination of higher levels of ApoE, C3, and HCFII together with lower ApoM being reported here; these dysregulated proteins may circumvent the protective effect of HDL and contribute to a more atherogenic profile that may increase cardiovascular risk and, at least in part, explain the atherogenic, inflammatory, insulin-resistant, and prothrombotic characteristics of PCOS. To mitigate these adverse risk factors, lifestyle modification including dietary modification and exercise should be emphasized for the management of women with PCOS with further interventional studies needed to determine if these findings are reversible.

## Figures and Tables

**Table 1:** Demographics, baseline hormonal, and metabolic parameters of the polycystic ovary syndrome (PCOS) subjects and controls.

Baseline Demographics	PCOS (n = 137)	Controls (n = 97)	*p*-Value
	Mean (SD)	Mean (SD)	
Age (years)	29.1 (6.1)	29.6 (6.5)	0.09
BMI (Kg/m^2^)	34.1 (7.5)	26.7 (6.6)	<0.0001
Body weight (Kg)	96.5 (23.7)	74.4 (18.4)	<0.0001
Insulin (IU/mL)	10.2 (6.1)	6.2 (3.2)	0.001
HOMA-IR	3.8 (0.6)	1.6 (0.2)	<0.005
CRP (mg/L)	4.4 (4.2)	2.4 (3.9)	0.0008
SHBG (nmol/L)	42.5 (39.6)	77.5 (78.4)	0.0003
Testosterone (nmol/l)	1.6 (1.0)	1.05 (0.48)	<0.0001
Total cholesterol (mmol/L)	4.8 (1.0)	4.7 (0.8)	0.22
Triglycerides (mmol/L)	1.5 (1.3)	1.0 (0.7)	0.001
HDL-cholesterol (mmol/L)	1.2 (0.3)	1.5 (0.4)	<0.0001
LDL-cholesterol (mmol/L)	2.9 (1.0)	2.7 (0.6)	0.16
TG/HDL ratio	1.4 (1.6)	0.8 (0.7)	0.001

BMI—body mass index; HOMA-IR—homeostasis model of assessment—insulin resistance; CRP—C-reactive protein; SHBG—sex hormone binding globulin; HDL-C—high-density lipoproteins cholesterol; LDL-C—low-density lipoproteins cholesterol.

## Data Availability

All the data for this study will be made available upon reasonable request to the corresponding author.

## Supplementary Materials

The following supporting information can be downloaded at https://www.mdpi.com/article/10.3390/cells12060855/s1. Supplementary Table S1. Protein levels measured by SOMAscan. Levels of proteins involved in lipid metabolism in subjects with polycystic ovary syndrome (PCOS) fulfilling all three diagnostic criteria versus controls. Data presented as Mean ± 1 Standard Deviation of Relative Fluorescent Units (RFU).
