# Supplementary material for: HDL-Associated Proteins in Subjects with Polycystic Ovary Syndrome: A Proteomic Study
Source: Cells. 2023 Mar 9;12(6):855. doi: 10.3390/cells12060855 (PMC10047209; doi:10.3390/cells12060855)
Supplement: Supplementary file 1 [file cells-12-00855-s001.zip › cells-2230943-supplementary.pdf]

Supplementary table S1. Levels of proteins involved in lipid metabolism in subjects with polycystic ovary syndrome (PCOS) fulfilling all three diagnostic criteria versus controls. Data presented as Mean ± 1 Standard Deviation of Relative Fluorescent Units (RFU).

	PCOS	Control	p value
Alpha-1-antichymotrypsin	248,880 (29,961)	244,203 (35,228)	0.27
Alpha-1-antitrypsin	1114 (371)	1213 (699)	0.15
Apolipoprotein A-I	14,631 (3465)	15,032 (2507)	0.33
Apolipoprotein B	11,213 (5408)	10,077 (3582)	0.07
Apolipoprotein D	3460 (693)	3371 (733)	0.34
Apolipoprotein E	39,054 (17,973)	33,577 (13,945)	0.012
Apolipoprotein E2	261,934 (50,357)	259,099 (51,596)	0.67
Apolipoprotein E3	217,377 (67,477)	201,576 (61,141)	0.06
Apolipoprotein E4	219,789 (58,305)	210,604 (55,689)	0.22
Apolipoprotein L1	41,476 (11,107)	41,089 (10,343)	0.79
Apolipoprotein M	7878 (3039)	9868 (3277)	0.000002
Clusterin	887 (151)	897 (253)	0.69
Complement C3	71,028 (25,536)	63,896 (26,822)	0.037
Hemopexin	1229 (623)	1167 (597)	0.44
Heparin cofactor II	4156 (773)	3821 (618)	0.0004
Kininogen-1	27,717 (6477)	27,501 (6331)	0.80
Serum amyloid A-1	1456 (2145)	1750 (4703)	0.51
Amyloid beta A4	27,043 (19,245)	23,874 (15,735)	0.18
Paraoxonase 1	151 (30)	146 (35)	0.24
